# Neutralizing antibody responses to the Delta variant of SARS-CoV-2 following vaccination with Ad5-nCoV (CanSino) in the Mexican population

**DOI:** 10.1371/journal.pone.0299520

**Published:** 2024-04-04

**Authors:** Jorge Hernández-Bello, Ana C. Lorenzo-Leal, José F. Muñoz-Valle, José J. Morales-Núñez, Saul A. Díaz-Pérez, Rodolfo Hernández-Gutiérrez, Horacio Bach

**Affiliations:** 1 Instituto de Investigación en Ciencias Biomédicas, Centro Universitario de Ciencias de la Salud Universidad de Guadalajara, Guadalajara, Mexico; 2 Division of Infectious Diseases, Faculty of Medicine, The University of British Columbia, Vancouver, BC, Canada; 3 Centro de Investigación y Asistencia en Tecnología y Diseño del Estado de Jalisco, Jalisco, Mexico; King Saud University College of Medicine, SAUDI ARABIA

## Abstract

During the COVID-19 pandemic, the Ad5-nCoV vaccine was applied to the Mexican population before the WHO approved it. In a transversal study, we compare the CanSino vaccine efficacy and a natural SARS-CoV-2 infection in eliciting neutralizing antibodies against the SARS-CoV-2 Delta variant in Guadalajara, Mexico. Participants between 30–60 years were included in the study and classified into three groups: 1) Natural immunity (unvaccinated), 2) Vaccine-induced immunity (vaccinated individuals without a COVID-19 history), and 3) Natural immunity + vaccine-induced immunity. These groups were matched by age and gender. We assessed the ability of individuals’ serum to neutralize the Delta variant and compared the results of the different groups using a neutralization test followed by plaque-forming units. Results showed that 39% of individuals’ serum with a history of COVID-19 (natural immunity, Group 1) could not neutralize the Delta variant, compared to 33% in vaccinated individuals without COVID-19 (vaccine immunity, Group 2). In contrast, only 7% of vaccinated individuals with a history of COVID-19 (natural + vaccine immunities) could not neutralize the Delta variant. We concluded that the effectiveness of the Ad5-nCoV vaccine to induce neutralizing antibodies against the Delta variant is comparable to that of natural infection (61% *vs*. 67%). However, in individuals with both forms of immunity (Group 3), it increased to 93%. Based on these results, despite the Ad5-nCoV vaccine originally being designed as a single-dose regimen, it could be recommended that even those who have recovered from COVID-19 should consider vaccination to boost their immunity against this variant.

## Introduction

The COVID-19 pandemic continues to pose a significant health challenge. Since its inception, the global scientific community has embarked on an unprecedented race to develop safe and effective vaccines to curtail the virus’s spread and impact [1].

Among the vaccines developed, the Ad5-nCoV vaccine (CanSino Biologics) was one of the prominent options deployed in China and some Latin American countries, including Mexico. Remarkably, Mexico began administering this vaccine before it received approval from the World Health Organization (WHO) [2]. This decision, driven by the dire need to protect each population amid rising COVID-19 cases, was not without controversy. In this context, the real-world evaluation of the Ad5-nCoV vaccine is still critical, as these data can inform future decision-making and enhance the resilience of our global health system. Therefore, it is paramount to understand its efficacy against variants like Delta (prominent in Mexico during this vaccine was applied) [3]. In 2021, the Delta variant not only heightened the likelihood of COVID-19 patients being hospitalized but also increased the mortality risk among individuals who had not received the vaccine [4].

A study reported that SARS-CoV-2 breakthrough infections during the Delta wave in a hospital from Monterrey, Mexico, mainly occurred in patients immunized with the Ad5-nCoV vaccine. Therefore, they concluded that SARS-CoV-2 variants compromised the efficacy of this vaccine [5].

Even though the Omicron variant has now displaced Delta and other variants worldwide [6], it remains crucial to understand the efficacy of the Ad5-nCoV vaccine against the Delta variant. This is particularly significant considering that the Delta variant was a predominant strain, and no studies were conducted on this combination of vaccine and variant during the height of the Delta variant’s prevalence. A broader understanding of the vaccine’s effectiveness against different virus strains is obtained by filling this knowledge gap. Such insights could provide valuable retrospective analysis, offer potential predictors for vaccine performance against future variants, and guide the process of vaccine modification if necessary.

Furthermore, the landscape of SARS-CoV-2 variants continues to evolve, and a resurgence of the Delta variant or new variants with similar characteristics cannot be completely ruled out. Hence, assessing the efficacy of available vaccines against different variants remains a crucial component of our global pandemic response strategy [7].

## Materials and methods

### Participants

In this study, 106 individuals from Guadalajara, Jalisco, Mexico were included and classified into three groups: 1) 46 unvaccinated individuals with a history of COVID-19 (“natural immunity, 1–3 months before sample collection), 2) 30 individuals vaccinated with a COVID-19 history (1–3 months before vaccination), and 30 individuals vaccinated without a COVID-19 history. These groups were matched by age and gender; vaccinated individuals were immunized with the Ad5-nCoV vaccine (CanSino Biologics Inc.). Before participating in the study, all recruited individuals provided their informed consent. This study complied with the principles outlined in the Declaration of Helsinki and received approval from the Committee of Ethics and Biosecurity of the CUCS, Universidad de Guadalajara, Mexico (Registry number 21–10).

Participants included in the study had a confirmed diagnosis of COVID-19 through RT-PCR testing conducted 1–3 months prior. All individuals experienced mild symptoms such as fever, cough, malaise, odynophagia, and headache, with no shortness of breath. Their oxygen saturation (SaO_2_) levels were above 94%, and their respiratory rate (R.R.) was below 20 breaths/min. To verify the absence of a previous COVID-19 history, the presence of anti-SARS-CoV-2 IgG/IgM antibodies was determined.

Additionally, to establish a seropositive threshold and evaluate the specificity of the plaque reduction neutralization test (PRNT), 14 negative control sera collected before the COVID-19 pandemic were subjected to testing.

Serum samples were obtained through venous puncture from May to August 2021 using Vacutainer tubes without anticoagulant at the Centro Universitario de Ciencias de la Salud (CUCS), Universidad de Guadalajara. A survey was administered to collect clinical and demographic information and the participants’ history of SARS-CoV-2 infection or vaccination.

### Laboratory test

#### Cells used in PRNT

To evaluate whether the Ad5-nCoV vaccine or natural infection-elicited antibodies can neutralize the Delta variant of SARS-CoV-2, we used PRNT.

Vero E6 cells (ATCC^®^ CRL-I1586^™^) were cultivated in MEM (Invitrogen) supplemented with 10% fetal bovine serum (Invitrogen), 1 mM sodium pyruvate (Invitrogen), non-essential amino acids (1X, Invitrogen), and 100 U/mL penicillin and 10 μg/mL gentamicin (Invitrogen) and used at passage < 40. Cells were cultivated following ATCC recommendations with 5% CO_2_ at 37°C. Cell density was kept below 2 × 10^6^ cells/mL, and cells were routinely tested for mycoplasma presence.

#### SARS-COV-2 infection

Following UBC and the Public Health Agency of Canada regulations, infections were performed in a Biosafety Level 3 (BSL3) at the Facility for Infectious Disease and Epidemic Research (FINDER) at UBC Vancouver. BCCDC provided the SARS-CoV-2 Delta variant. Viral stocks were propagated in Vero E6 cells, harvested, and serial dilutions were performed to calculate the final titer. For experiments, a viral stock at 5.9 × 10^6^ plaque-forming units (PFU)/mL) was used. Vero E6 cells were seeded at 4 × 10^5^ cells/well in clear, flat bottom 12-well plates the day before infection.

Sera were diluted in OptiMEM (Invitrogen) in a range of 1:10–1:1280 and supplemented with 10 μL of the virus (5.9 x 10^4^) using 96-well plates. The mixture was mixed for 1 h using a nutator rocker. Then, the supernatants of the cells were disposed of, and the total volume (100 μL) of each reaction was used to overlay the cells for 1 h, with gentle rotation every 15 min at 37°C supplemented with 5% CO_2_. The 100 μL containing the virus were disposed of, and the cells were overlayed with 1 mL MEM (2X) supplemented with 1 mL 2% colloidal cellulose (Sigma) and returned to the incubator for 72 h.

#### SARS-COV-2 plaque assay

After 72 h post-infection, the overlaying media was disposed of, and 1 mL paraformaldehyde (4%) was added to each well for 30 min. Then, the paraformaldehyde was collected, and 200 μL of 1% crystal violet (in 20% methanol) was added to each well for 10 min. Then, cells were washed with 1 mL tap water/well trice, air-dried, and plaques were counted by visualization. To establish a seropositive threshold and evaluate the specificity of the PRNT test, 14 negative control sera collected before the COVID-19 pandemic were subjected to testing.

#### Statistical analyses

Data analysis was performed by the GraphPad Prism version 8.4.1 for Windows (GraphPad Software, LLC) and the R software (version 4.0.1., R Development Core Team). The level of statistical significance was set at *p* < 0.05. Non-parametric tests (Kruskall-Walli’s test) were used to compare data expressed in medians between groups. Frequencies of neutralizing antibody titers were compared by chi-square test.

## Results

### Sociodemographic data

The sociodemographic characteristics of individuals of each group are described in Table 1. No significant differences were found in gender frequency, age, treatment, and comorbidities between them (*p* >0.05).

**Table 1:** Sociodemographic data from study groups.

Characteristics	Group 1	Group 2	Group 3	*p*-value
Gender^1^				
Male	21 (46%)	15 (50%)	15 (50%)	0.9
Female	25 (54%)	15 (50%)	15 (50%)	
Age^2^	43 (33–54)	41 (28–58)	42 (32–57)	0.9
Comorbidity presence^1^	20 (43%)	15 (50%)	17 (57%)	0.5
Treatment	21 (46%)	15 (50%)	8 (27%)	0.1
Use of treatment for symptoms^1^	17 (45%)	6 (27%)	12 (43%)	0.4

Group 1, Natural immunity (unvaccinated); Group 2, Vaccine-induced immunity (vaccinated individuals, without a COVID-19 history); Group 3, Natural immunity + vaccine-induced immunity.

^1^ n (%), Chi-squared tests, Median (IQR), Kruskal-Walli’s test

^2^ Mann-Whitney’s U test, Fisher’s exact test.

### Neutralizing responses

A PRNT was utilized to evaluate whether the serum of the evaluated individuals could neutralize the Delta variant of SARS-CoV-2. All 14 negative control sera sampled failed to neutralize the Delta variant (titers < 1:10); therefore, test sera recording titers equal to or above 1:10 were considered positive.

**Fig 1:**
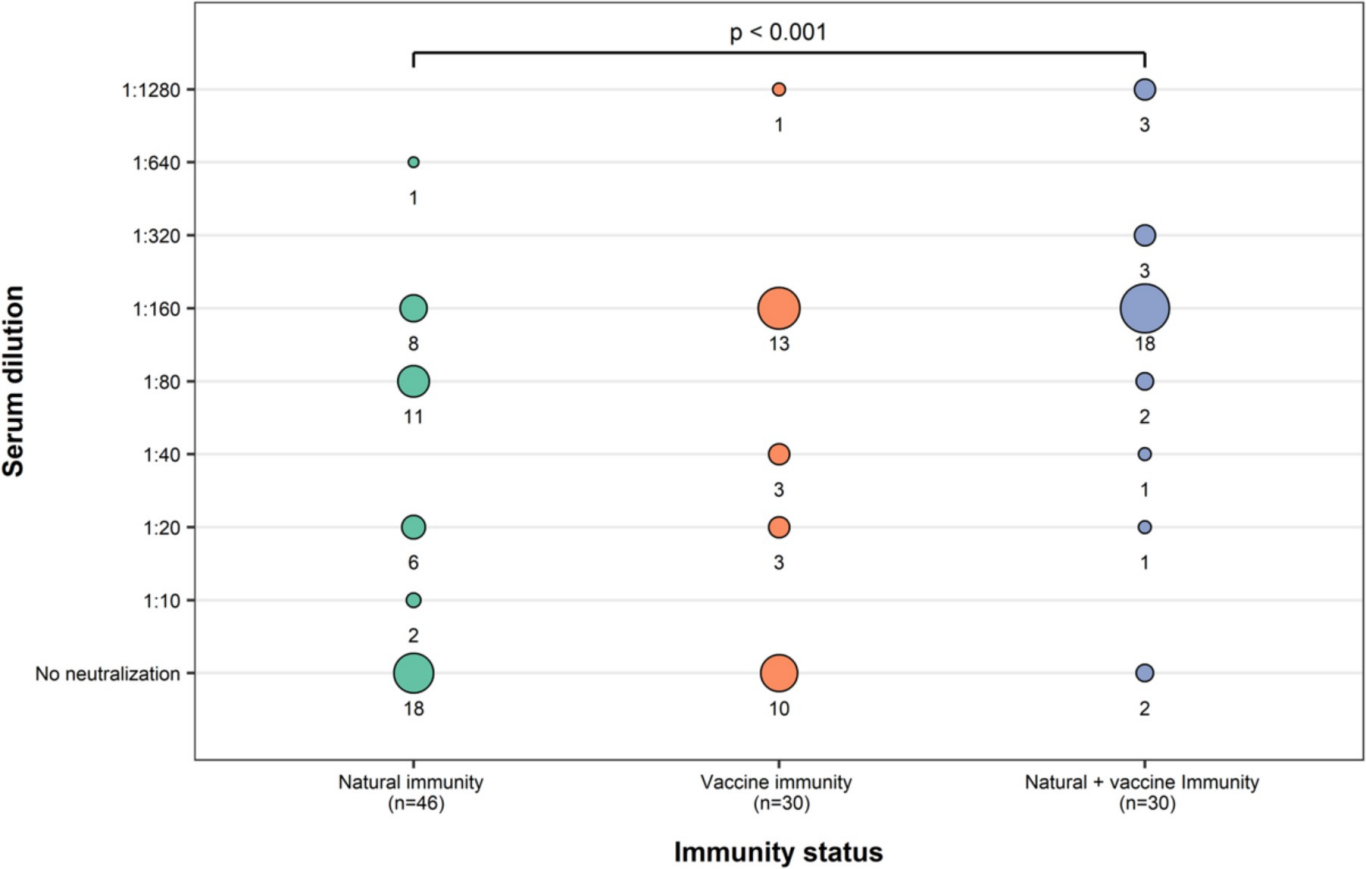
Comparison of Delta variant neutralization under natural immunity or vaccine-induced immunity. The chi-square test was used for statistical differences in the groups. Sera from 18 (39%) individuals with a history of COVID-19 (natural immunity) could not neutralize the Delta variant. In contrast, serum from 10 (33%) vaccinated individuals without a history of COVID-19 (vaccine immunity) could not neutralize the Delta variant. Concerning sera from vaccinated individuals with a history of COVID-19 (natural immunity + vaccine immunity), only 2 (7%) could not neutralize the Delta variant.

The frequency of neutralization in each dilution for the other three groups is illustrated in Fig 1. Panel A presents data on COVID-19 individuals 21 days after infection; Panel B shows neutralization after 21 days post-COVID-19 vaccination in individuals without COVID-19 history, while panel C displays neutralization after 21 days post-COVID-19 vaccination in individuals with COVID-19 history.

A significant difference was observed between subgroups when comparing neutralization levels (dilutions) (*p*<0.001), with an increase in virus neutralization in vaccinated individuals with a COVID-19 history.

In the group with natural immunity, only 32% of them (9/28) had neutralizing titers ≥ 1:160, while it was 70% (14/20) in those immunized with Ad5-nCoV without a COVID-19 history (vaccine immunity). In contrast, 86% (24/28) of individuals with prior COVID-19 infection + Ad5-nCoV immunization had neutralizing titles ≥ 1:160.

### Comparison of logarithmic reduction

**Fig 2:**
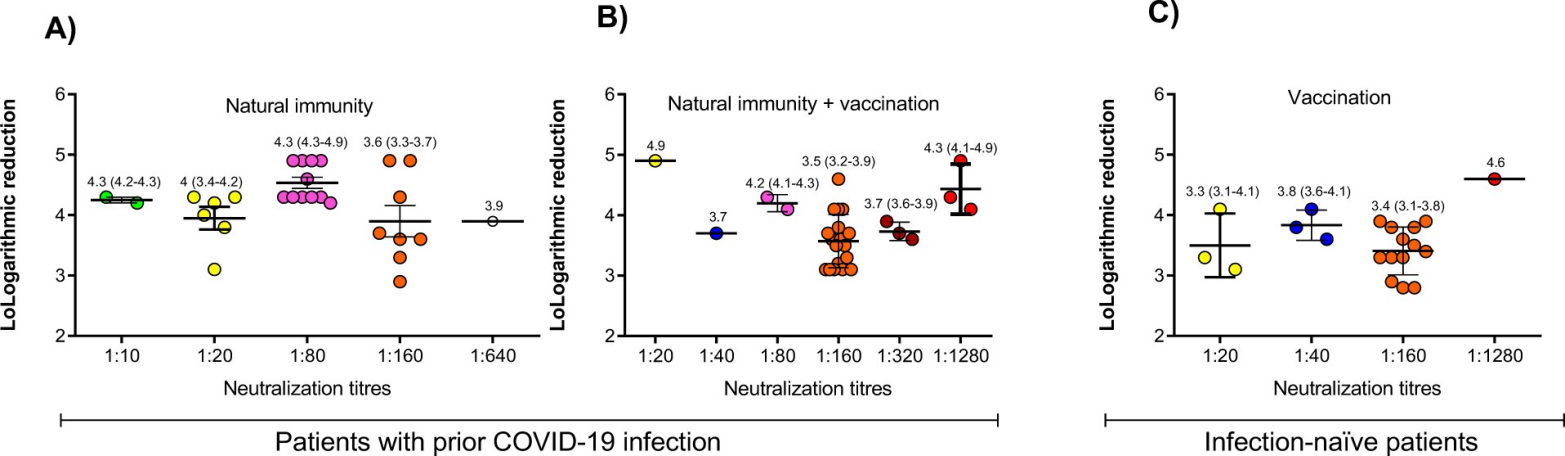
The median and interquartile range of logarithm reduction over the different serum dilutions. The effects of the vaccination on the neutralization of group (A) natural infection, (B) natural infection + immunization, and (C) vaccination are shown.

The logarithmic reduction was also reported for each serum dilution, with a higher value indicating higher neutralization. The median and interquartile range from every dilution is indicated in Fig 2.

In individuals without prior SARS-CoV-2 infection, those vaccinated who did generate neutralizing antibodies against the Delta variant (Fig 2C) show similar logarithm reductions in each neutralization titer than those generated under natural immunity (Fig 2A). However, both groups had lower logarithm reductions than those individuals in the natural immunity + vaccination group (Fig 2B).

## Discussion

Our study offers preliminary insights into the effectiveness of the Ad5-nCoV vaccine against the Delta variant of SARS-CoV-2, particularly within a specific subset of the Mexican population.

The Ad5-nCoV vaccine received emergency use authorization in eight countries, including Argentina, Chile, China, Ecuador, Hungary, Malaysia, Pakistan, and Mexico [8]. This vaccine is currently administered as a single dose based on a previous study demonstrating robust immune responses in most recipients following a single intramuscular immunization. Furthermore, it produces neutralizing antibodies that effectively target live SARS-CoV-2 [9].

Recently, the Phase 3 trial of the Ad5-nCoV vaccine analyzing different countries worldwide, including Mexico, revealed that this vaccine exhibited 57.5% efficacy against symptomatic, PCR-confirmed COVID-19 infection 28 days post-vaccination. Moreover, efficacy against severe disease was 91.7% (95% CI 36.1 to 99.0) beginning 28 days post-vaccination and 96% (95% CI 70.5 to 99.5) beginning 14 days post-vaccination [10]. However, it is crucial to consider that these trials were conducted predominantly when the original strain of the virus was circulating.

When comparing different types of vaccines, it becomes evident that Ad5-nCoV exhibits lower efficacy than mRNA vaccines. Nevertheless, it demonstrates a higher effectiveness in generating neutralizing antibodies when compared to other viral vector vaccines, such as the Ad26.COV2.S/Janssen [11, 12].

The observation that 33% of individuals immunized with Ad5-nCoV could not neutralize the Delta variant echoes other studies indicating the limitations of this vaccine against emerging SARS-CoV-2 variants. This aligns with another research, which found that neutralizing antibody titers against the Delta variant were lower in individuals vaccinated with adenovirus vector-based vaccine (such as Ad5-nCoV) than those vaccinated with mRNA vaccines [13]. Also, these data can support a previous study in the Mexican population reporting that SARS-CoV-2 breakthrough infections during the Delta wave in a Mexican hospital mainly occurred in patients vaccinated with the Ad5-nCoV vaccine compared to those vaccinated with other platforms. Moreover, they suggested that a booster dose of the Ad5-nCoV vaccine is advisable for those individuals without previous infection to improve their immune response against SARS-CoV-2 [14].

In addition to the above, we also observe that only 70% of the vaccinated individuals without a COVID-19 history (vaccine immunity group) showed titers ≥ 1:160, which again raises concerns about the overall effectiveness of the vaccination alone. This is based on the minimum titer recommended by the FDA for neutralizing the activity of convalescent plasma administered in randomized controlled trials of COVID‐19 infection [15, 16]. However, in those who had been infected and then immunized (with the Ad5-nCoV vaccine), 86% had neutralizing titers ≥ 1:160. This underlines the importance of continued research and optimization of vaccination strategies to ensure robust immunity across the population.

According to the search performed, there are no previous reports evaluating the ability of the antibodies induced by this vaccine to neutralize the Delta variant. However, since this vaccine was made against the S protein of the original variant of SARS-CoV-2 [17], this can be extrapolated from a previous study that demonstrated that immunity induced by the original strain of SARS-CoV-2 had reduced efficacy against the Delta variant. This has been associated with critical mutations of this variant, such as D614G, L452R, P681R, and T478K in the S-protein [18, 19]. Thus, there is a need to complement the single vaccination scheme proposed for this vaccine or consider a heterologous vaccination with another vaccine with greater efficacy against this variant.

Our data highlight the importance of “hybrid immunity,” combining both natural immunities from previous infection and vaccine-induced immunity, as we corroborated different immune responses to vaccination between individuals with and without a history of COVID-19, aligning with previously conducted research [20]. This also aligns with other recent studies showing that individuals with a history of SARS-CoV-2 infection who receive vaccination tended to generate robust neutralizing responses against different virus variants [21]. In a previous study cohort, we specifically examined the effects of heterologous vaccination using the Ad5-nCoV vaccine, followed by a booster dose of ChAdOx1-S-Nov-19, Ad26.COV2.S, BNT162b2, or mRNA-127. Our findings indicate that this heterologous vaccination approach is well-tolerated and elicits a more robust humoral immune response [22].

The Ad5-nCoV vaccine is currently approved in 10 Latin American, Asian, and European countries; however, little information is available about its effectiveness [23]. Our findings underline the need for continued monitoring of vaccine efficacy against emerging variants and potentially adjusting vaccination strategies, such as booster shots, to ensure optimal protection.

Despite the observational nature of this study and the relatively small cohort size, our results provide a critical understanding of the Ad5-nCoV vaccine’s real-world effectiveness against the Delta variant. The study’s limited enrollment of 106 patients stemmed from specific challenges in recruiting individuals in Guadalajara, Jalisco, Mexico, who had been vaccinated with the Ad5-nCoV vaccine, initially targeted exclusively at teachers. The vaccine’s lack of WHO approval at that time generated significant hesitancy and resistance among potential participants. In response, an informative campaign at the University of Guadalajara was initiated to encourage study participation among those vaccinated in the government’s campaign. This specific focus and prevailing hesitancy restricted the available participant pool. Additionally, the study ensured that the participants’ prior COVID-19 infection or vaccination timeline, age, and sex were representative of the vaccinated population. Future research on a larger scale is essential to verify these results and further assess the vaccine’s efficacy against various viral variants, as well as the durability of the immune response.

## Supporting information

S1 File (XLSX)
